# Supplementary material for: Modeling glioblastoma heterogeneity as a dynamic network of cell states
Source: Mol Syst Biol. 2021 Sep 16;17(9):e10105. doi: 10.15252/msb.202010105 (PMC8444284; doi:10.15252/msb.202010105)
Supplement: Supplementary file 6 — Source Data for Figure 5 [file MSB-17-e10105-s004.zip › Figure5A_sourcedata/GSEA_3071/nosigdb_stateII.GseaPreranked.1622795151171/RICHARDS_NOWAKOWSKI_SCIENCE_2017_VRG_UPREG.html]

Details for gene set RICHARDS\_NOWAKOWSKI\_SCIENCE\_2017\_VRG\_UPREG[GSEA]

|  || Dataset | state23071 |
| Phenotype | NoPhenotypeAvailable |
| Upregulated in class | na\_neg |
| GeneSet | RICHARDS\_NOWAKOWSKI\_SCIENCE\_2017\_VRG\_UPREG |
| Enrichment Score (ES) | -0.48916808 |
| Normalized Enrichment Score (NES) | -3.0956807 |
| Nominal p-value | 0.0 |
| FDR q-value | 0.0 |
| FWER p-Value | 0.0 |
Table: GSEA Results Summary

  

Fig 1: Enrichment plot: RICHARDS\_NOWAKOWSKI\_SCIENCE\_2017\_VRG\_UPREG      
 Profile of the Running ES Score & Positions of GeneSet Members on the Rank Ordered List

  

| PROBE | GENE SYMBOL | GENE\_TITLE | RANK IN GENE LIST | RANK METRIC SCORE | RUNNING ES | CORE ENRICHMENT || 1 | F3 |  |  | 13 | 1.555 | 0.0069 | No |
| 2 | AXL |  |  | 129 | 0.997 | -0.0163 | No |
| 3 | MYL12A |  |  | 174 | 0.910 | -0.0216 | No |
| 4 | ADAMTS6 |  |  | 191 | 0.884 | -0.0200 | No |
| 5 | ACTN1 |  |  | 256 | 0.813 | -0.0311 | No |
| 6 | CYR61 |  |  | 267 | 0.791 | -0.0285 | No |
| 7 | VCL |  |  | 278 | 0.778 | -0.0260 | No |
| 8 | BARD1 |  |  | 295 | 0.760 | -0.0251 | No |
| 9 | AKAP12 |  |  | 349 | 0.711 | -0.0341 | No |
| 10 | NRG1 |  |  | 356 | 0.708 | -0.0310 | No |
| 11 | KLF6 |  |  | 357 | 0.708 | -0.0264 | No |
| 12 | MT2A |  |  | 393 | 0.682 | -0.0309 | No |
| 13 | ZFP36L2 |  |  | 458 | 0.647 | -0.0432 | No |
| 14 | EZR |  |  | 466 | 0.642 | -0.0408 | No |
| 15 | LRIG3 |  |  | 493 | 0.630 | -0.0433 | No |
| 16 | FSTL1 |  |  | 495 | 0.630 | -0.0394 | No |
| 17 | ENO1 |  |  | 501 | 0.629 | -0.0366 | No |
| 18 | PDLIM5 |  |  | 526 | 0.613 | -0.0388 | No |
| 19 | TMEM98 |  |  | 594 | 0.580 | -0.0523 | No |
| 20 | CACYBP |  |  | 613 | 0.573 | -0.0531 | No |
| 21 | WEE1 |  |  | 685 | 0.536 | -0.0679 | No |
| 22 | HOPX |  |  | 768 | 0.510 | -0.0858 | No |
| 23 | ANXA2 |  |  | 839 | 0.488 | -0.1006 | No |
| 24 | PLAGL1 |  |  | 892 | 0.473 | -0.1110 | No |
| 25 | TLN2 |  |  | 917 | 0.467 | -0.1141 | No |
| 26 | TAGLN2 |  |  | 940 | 0.461 | -0.1167 | No |
| 27 | CSMD2 |  |  | 966 | 0.454 | -0.1202 | No |
| 28 | SOAT1 |  |  | 982 | 0.449 | -0.1211 | No |
| 29 | DDAH1 |  |  | 1028 | 0.434 | -0.1299 | No |
| 30 | NOG |  |  | 1031 | 0.433 | -0.1276 | No |
| 31 | JADE1 |  |  | 1089 | 0.419 | -0.1395 | No |
| 32 | TPI1 |  |  | 1111 | 0.415 | -0.1422 | No |
| 33 | ITGA2 |  |  | 1114 | 0.415 | -0.1400 | No |
| 34 | NOTCH3 |  |  | 1141 | 0.409 | -0.1440 | No |
| 35 | CNTLN |  |  | 1142 | 0.409 | -0.1413 | No |
| 36 | COL11A1 |  |  | 1163 | 0.404 | -0.1438 | No |
| 37 | NBPF9 |  |  | 1172 | 0.403 | -0.1433 | No |
| 38 | DHCR24 |  |  | 1253 | 0.386 | -0.1614 | No |
| 39 | YBX3 |  |  | 1301 | 0.378 | -0.1710 | No |
| 40 | EFNB2 |  |  | 1306 | 0.377 | -0.1696 | No |
| 41 | STK17B |  |  | 1324 | 0.374 | -0.1715 | No |
| 42 | HSDL2 |  |  | 1326 | 0.373 | -0.1693 | No |
| 43 | GLUD1 |  |  | 1397 | 0.358 | -0.1850 | No |
| 44 | CAPN2 |  |  | 1426 | 0.352 | -0.1899 | No |
| 45 | PSAT1 |  |  | 1505 | 0.340 | -0.2079 | No |
| 46 | HSPH1 |  |  | 1595 | 0.324 | -0.2287 | No |
| 47 | PHACTR2 |  |  | 1596 | 0.324 | -0.2266 | No |
| 48 | TMX1 |  |  | 1703 | 0.310 | -0.2519 | No |
| 49 | SLC12A2 |  |  | 1712 | 0.309 | -0.2519 | No |
| 50 | ATP2B4 |  |  | 1713 | 0.308 | -0.2499 | No |
| 51 | JUN |  |  | 1725 | 0.307 | -0.2507 | No |
| 52 | PUS7 |  |  | 1741 | 0.306 | -0.2526 | No |
| 53 | VCAN |  |  | 1793 | 0.299 | -0.2638 | No |
| 54 | PALLD |  |  | 1820 | 0.297 | -0.2686 | No |
| 55 | IRF2BP2 |  |  | 1844 | 0.293 | -0.2726 | No |
| 56 | TSPAN5 |  |  | 1861 | 0.291 | -0.2748 | No |
| 57 | GLO1 |  |  | 1867 | 0.290 | -0.2742 | No |
| 58 | STK39 |  |  | 1871 | 0.289 | -0.2731 | No |
| 59 | CPT1A |  |  | 1959 | 0.278 | -0.2937 | No |
| 60 | FERMT2 |  |  | 2003 | 0.272 | -0.3030 | No |
| 61 | RCN1 |  |  | 2051 | 0.267 | -0.3134 | No |
| 62 | LDLR |  |  | 2128 | 0.260 | -0.3313 | No |
| 63 | EIF2AK2 |  |  | 2148 | 0.257 | -0.3345 | No |
| 64 | ZDHHC13 |  |  | 2158 | 0.256 | -0.3352 | No |
| 65 | ANXA5 |  |  | 2187 | 0.253 | -0.3407 | No |
| 66 | HSPA4L |  |  | 2203 | 0.252 | -0.3429 | No |
| 67 | PEA15 |  |  | 2209 | 0.251 | -0.3426 | No |
| 68 | NRBP2 |  |  | 2271 | -0.256 | -0.3567 | No |
| 69 | LRP4 |  |  | 2300 | -0.259 | -0.3622 | No |
| 70 | SNX13 |  |  | 2329 | -0.262 | -0.3677 | No |
| 71 | MSI1 |  |  | 2379 | -0.268 | -0.3786 | No |
| 72 | PPP2CB |  |  | 2394 | -0.270 | -0.3804 | No |
| 73 | DTNA |  |  | 2414 | -0.272 | -0.3835 | No |
| 74 | CANX |  |  | 2427 | -0.274 | -0.3848 | No |
| 75 | CDON |  |  | 2454 | -0.278 | -0.3897 | No |
| 76 | SPRY2 |  |  | 2455 | -0.278 | -0.3879 | No |
| 77 | STX16 |  |  | 2468 | -0.279 | -0.3892 | No |
| 78 | ZDHHC2 |  |  | 2474 | -0.280 | -0.3886 | No |
| 79 | SEPT2 |  |  | 2482 | -0.281 | -0.3886 | No |
| 80 | MID1 |  |  | 2486 | -0.283 | -0.3875 | No |
| 81 | CNTNAP3B |  |  | 2490 | -0.283 | -0.3864 | No |
| 82 | GATM |  |  | 2501 | -0.285 | -0.3871 | No |
| 83 | SRGAP3 |  |  | 2508 | -0.286 | -0.3868 | No |
| 84 | EMC2 |  |  | 2512 | -0.287 | -0.3857 | No |
| 85 | SIPA1L1 |  |  | 2547 | -0.293 | -0.3925 | No |
| 86 | PDCD10 |  |  | 2556 | -0.294 | -0.3926 | No |
| 87 | HMGN3 |  |  | 2561 | -0.295 | -0.3917 | No |
| 88 | GPC4 |  |  | 2577 | -0.298 | -0.3936 | No |
| 89 | DIO2 |  |  | 2599 | -0.301 | -0.3971 | No |
| 90 | TMEM132B |  |  | 2623 | -0.304 | -0.4010 | No |
| 91 | SRGAP2C |  |  | 2628 | -0.305 | -0.4001 | No |
| 92 | EVI5 |  |  | 2635 | -0.306 | -0.3996 | No |
| 93 | NES |  |  | 2803 | -0.335 | -0.4405 | No |
| 94 | METTL7A |  |  | 2818 | -0.339 | -0.4419 | No |
| 95 | SPAG16 |  |  | 2822 | -0.340 | -0.4404 | No |
| 96 | RYK |  |  | 2829 | -0.341 | -0.4397 | No |
| 97 | ANGPTL1 |  |  | 2840 | -0.343 | -0.4401 | No |
| 98 | PTPN13 |  |  | 2871 | -0.348 | -0.4455 | No |
| 99 | NPHP3 |  |  | 2899 | -0.355 | -0.4502 | No |
| 100 | SLITRK2 |  |  | 2970 | -0.368 | -0.4658 | No |
| 101 | SERF2 |  |  | 2975 | -0.368 | -0.4644 | No |
| 102 | JAM3 |  |  | 3016 | -0.378 | -0.4723 | No |
| 103 | APBB2 |  |  | 3023 | -0.378 | -0.4713 | No |
| 104 | ALDH6A1 |  |  | 3027 | -0.379 | -0.4696 | No |
| 105 | PHYHIPL |  |  | 3049 | -0.385 | -0.4725 | No |
| 106 | SCRN1 |  |  | 3051 | -0.386 | -0.4702 | No |
| 107 | SPRED1 |  |  | 3054 | -0.387 | -0.4682 | No |
| 108 | PAX6 |  |  | 3055 | -0.387 | -0.4657 | No |
| 109 | ABAT |  |  | 3075 | -0.391 | -0.4680 | No |
| 110 | PDCD6 |  |  | 3082 | -0.393 | -0.4670 | No |
| 111 | BMP7 |  |  | 3127 | -0.407 | -0.4756 | No |
| 112 | FBXO32 |  |  | 3149 | -0.413 | -0.4783 | No |
| 113 | NDUFB5 |  |  | 3158 | -0.415 | -0.4777 | No |
| 114 | CTNNA1 |  |  | 3163 | -0.418 | -0.4760 | No |
| 115 | TMED10 |  |  | 3179 | -0.423 | -0.4771 | No |
| 116 | FBN2 |  |  | 3186 | -0.425 | -0.4758 | No |
| 117 | HEG1 |  |  | 3200 | -0.429 | -0.4763 | No |
| 118 | NOTCH1 |  |  | 3202 | -0.429 | -0.4738 | No |
| 119 | PON2 |  |  | 3216 | -0.432 | -0.4743 | No |
| 120 | SOX9 |  |  | 3230 | -0.435 | -0.4748 | No |
| 121 | NPAS3 |  |  | 3233 | -0.437 | -0.4724 | No |
| 122 | TSPAN6 |  |  | 3240 | -0.438 | -0.4711 | No |
| 123 | SDCBP |  |  | 3257 | -0.442 | -0.4723 | No |
| 124 | HIGD1A |  |  | 3310 | -0.460 | -0.4827 | No |
| 125 | ITGA6 |  |  | 3315 | -0.461 | -0.4807 | No |
| 126 | FAM84A |  |  | 3344 | -0.474 | -0.4848 | No |
| 127 | CHP1 |  |  | 3354 | -0.479 | -0.4840 | No |
| 128 | RHOJ |  |  | 3375 | -0.488 | -0.4860 | Yes |
| 129 | SHROOM3 |  |  | 3387 | -0.493 | -0.4856 | Yes |
| 130 | NFE2L2 |  |  | 3389 | -0.493 | -0.4826 | Yes |
| 131 | MRC2 |  |  | 3391 | -0.494 | -0.4796 | Yes |
| 132 | FZD8 |  |  | 3399 | -0.498 | -0.4781 | Yes |
| 133 | DOK5 |  |  | 3409 | -0.503 | -0.4771 | Yes |
| 134 | APC |  |  | 3422 | -0.510 | -0.4769 | Yes |
| 135 | RFTN2 |  |  | 3427 | -0.511 | -0.4745 | Yes |
| 136 | ARX |  |  | 3443 | -0.516 | -0.4750 | Yes |
| 137 | SEMA6A |  |  | 3455 | -0.521 | -0.4744 | Yes |
| 138 | QKI |  |  | 3467 | -0.526 | -0.4738 | Yes |
| 139 | LRP6 |  |  | 3473 | -0.530 | -0.4716 | Yes |
| 140 | JAM2 |  |  | 3477 | -0.532 | -0.4689 | Yes |
| 141 | STAT3 |  |  | 3488 | -0.537 | -0.4679 | Yes |
| 142 | AGO3 |  |  | 3500 | -0.541 | -0.4672 | Yes |
| 143 | FABP5 |  |  | 3513 | -0.549 | -0.4667 | Yes |
| 144 | ITM2C |  |  | 3518 | -0.552 | -0.4641 | Yes |
| 145 | ARL6IP5 |  |  | 3529 | -0.557 | -0.4630 | Yes |
| 146 | AASS |  |  | 3539 | -0.564 | -0.4616 | Yes |
| 147 | INTU |  |  | 3540 | -0.565 | -0.4579 | Yes |
| 148 | FHOD3 |  |  | 3559 | -0.576 | -0.4588 | Yes |
| 149 | RHOQ |  |  | 3562 | -0.578 | -0.4555 | Yes |
| 150 | IFITM3 |  |  | 3578 | -0.589 | -0.4555 | Yes |
| 151 | PTGFRN |  |  | 3584 | -0.595 | -0.4529 | Yes |
| 152 | ZEB1 |  |  | 3589 | -0.598 | -0.4500 | Yes |
| 153 | DBI |  |  | 3597 | -0.601 | -0.4478 | Yes |
| 154 | BBX |  |  | 3600 | -0.605 | -0.4443 | Yes |
| 155 | PCMTD1 |  |  | 3601 | -0.605 | -0.4404 | Yes |
| 156 | DACH1 |  |  | 3614 | -0.613 | -0.4394 | Yes |
| 157 | AIF1L |  |  | 3621 | -0.617 | -0.4369 | Yes |
| 158 | PHLDA1 |  |  | 3622 | -0.617 | -0.4329 | Yes |
| 159 | CDCA7L |  |  | 3642 | -0.637 | -0.4336 | Yes |
| 160 | SHC3 |  |  | 3648 | -0.645 | -0.4306 | Yes |
| 161 | MEIS1 |  |  | 3651 | -0.648 | -0.4269 | Yes |
| 162 | MLC1 |  |  | 3658 | -0.652 | -0.4241 | Yes |
| 163 | SAT1 |  |  | 3659 | -0.653 | -0.4198 | Yes |
| 164 | DAG1 |  |  | 3662 | -0.654 | -0.4161 | Yes |
| 165 | SH3BGRL |  |  | 3663 | -0.654 | -0.4118 | Yes |
| 166 | LITAF |  |  | 3670 | -0.660 | -0.4090 | Yes |
| 167 | FGFR1 |  |  | 3672 | -0.662 | -0.4049 | Yes |
| 168 | GAB1 |  |  | 3684 | -0.670 | -0.4033 | Yes |
| 169 | MPPED2 |  |  | 3701 | -0.685 | -0.4029 | Yes |
| 170 | TTYH3 |  |  | 3712 | -0.694 | -0.4009 | Yes |
| 171 | PREX1 |  |  | 3716 | -0.698 | -0.3971 | Yes |
| 172 | TGIF1 |  |  | 3728 | -0.707 | -0.3953 | Yes |
| 173 | FAT1 |  |  | 3730 | -0.708 | -0.3909 | Yes |
| 174 | RALGAPA2 |  |  | 3732 | -0.710 | -0.3865 | Yes |
| 175 | HTRA1 |  |  | 3741 | -0.719 | -0.3838 | Yes |
| 176 | JUND |  |  | 3747 | -0.725 | -0.3804 | Yes |
| 177 | CTNNB1 |  |  | 3753 | -0.731 | -0.3768 | Yes |
| 178 | RAB31 |  |  | 3757 | -0.737 | -0.3728 | Yes |
| 179 | NCAN |  |  | 3763 | -0.748 | -0.3691 | Yes |
| 180 | FZD3 |  |  | 3770 | -0.761 | -0.3657 | Yes |
| 181 | SALL3 |  |  | 3778 | -0.768 | -0.3624 | Yes |
| 182 | TRPS1 |  |  | 3804 | -0.793 | -0.3637 | Yes |
| 183 | TTYH1 |  |  | 3807 | -0.795 | -0.3590 | Yes |
| 184 | BICD1 |  |  | 3814 | -0.801 | -0.3552 | Yes |
| 185 | MSI2 |  |  | 3816 | -0.802 | -0.3502 | Yes |
| 186 | MAGI1 |  |  | 3827 | -0.815 | -0.3474 | Yes |
| 187 | CHMP2A |  |  | 3859 | -0.850 | -0.3499 | Yes |
| 188 | SALL1 |  |  | 3863 | -0.855 | -0.3450 | Yes |
| 189 | PPP1CB |  |  | 3865 | -0.862 | -0.3396 | Yes |
| 190 | PSAP |  |  | 3882 | -0.907 | -0.3377 | Yes |
| 191 | SRI |  |  | 3885 | -0.909 | -0.3323 | Yes |
| 192 | DDR1 |  |  | 3903 | -0.948 | -0.3304 | Yes |
| 193 | FOS |  |  | 3911 | -0.966 | -0.3259 | Yes |
| 194 | SEL1L3 |  |  | 3919 | -1.001 | -0.3211 | Yes |
| 195 | ROBO1 |  |  | 3920 | -1.005 | -0.3145 | Yes |
| 196 | SEMA5A |  |  | 3921 | -1.006 | -0.3079 | Yes |
| 197 | STK17A |  |  | 3925 | -1.012 | -0.3020 | Yes |
| 198 | TRIM9 |  |  | 3930 | -1.016 | -0.2963 | Yes |
| 199 | ETV1 |  |  | 3934 | -1.026 | -0.2904 | Yes |
| 200 | SLC1A3 |  |  | 3942 | -1.040 | -0.2853 | Yes |
| 201 | TRIB2 |  |  | 3944 | -1.041 | -0.2787 | Yes |
| 202 | SOX2 |  |  | 3953 | -1.069 | -0.2738 | Yes |
| 203 | PTPRG |  |  | 3958 | -1.083 | -0.2677 | Yes |
| 204 | ITGB8 |  |  | 3962 | -1.093 | -0.2613 | Yes |
| 205 | ZFHX4 |  |  | 3963 | -1.095 | -0.2541 | Yes |
| 206 | ETV5 |  |  | 3971 | -1.116 | -0.2485 | Yes |
| 207 | MYO10 |  |  | 3990 | -1.193 | -0.2453 | Yes |
| 208 | RGS16 |  |  | 3996 | -1.227 | -0.2385 | Yes |
| 209 | FGFBP3 |  |  | 3999 | -1.237 | -0.2309 | Yes |
| 210 | ZBTB20 |  |  | 4024 | -1.407 | -0.2279 | Yes |
| 211 | SLC35F1 |  |  | 4035 | -1.515 | -0.2205 | Yes |
| 212 | TNC |  |  | 4039 | -1.549 | -0.2111 | Yes |
| 213 | SLC1A2 |  |  | 4040 | -1.555 | -0.2008 | Yes |
| 214 | MOXD1 |  |  | 4045 | -1.591 | -0.1914 | Yes |
| 215 | PDPN |  |  | 4046 | -1.612 | -0.1808 | Yes |
| 216 | SYT11 |  |  | 4047 | -1.617 | -0.1702 | Yes |
| 217 | CLU |  |  | 4069 | -1.908 | -0.1630 | Yes |
| 218 | MEIS2 |  |  | 4073 | -2.041 | -0.1504 | Yes |
| 219 | CKB |  |  | 4075 | -2.149 | -0.1365 | Yes |
| 220 | PTN |  |  | 4077 | -2.187 | -0.1224 | Yes |
| 221 | NFIA |  |  | 4078 | -2.210 | -0.1078 | Yes |
| 222 | ID4 |  |  | 4079 | -2.308 | -0.0926 | Yes |
| 223 | GFAP |  |  | 4080 | -2.343 | -0.0772 | Yes |
| 224 | GPM6B |  |  | 4083 | -2.435 | -0.0617 | Yes |
| 225 | C1orf61 |  |  | 4084 | -2.474 | -0.0454 | Yes |
| 226 | FABP7 |  |  | 4095 | -3.351 | -0.0260 | Yes |
| 227 | PTPRZ1 |  |  | 4097 | -4.027 | 0.0003 | Yes |
Table: GSEA details [plain text format]

  

Fig 2: RICHARDS\_NOWAKOWSKI\_SCIENCE\_2017\_VRG\_UPREG: Random ES distribution      
 Gene set null distribution of ES for **RICHARDS\_NOWAKOWSKI\_SCIENCE\_2017\_VRG\_UPREG**
